# Laxative effects of agarwood on low-fiber diet-induced constipation in rats

**DOI:** 10.1186/1472-6882-10-68

**Published:** 2010-11-15

**Authors:** Mamoru Kakino, Shigemi Tazawa, Hiroe Maruyama, Kazuhiro Tsuruma, Yoko Araki, Masamitsu Shimazawa, Hideaki Hara

**Affiliations:** 1 Molecular Pharmacology, Department of Biofunctional Evaluation, Gifu Pharmaceutical University, Gifu 501-1196, Japan; 2 Nagara Research Center, API Co., Ltd., 692-3 Nagarayamasaki, Gifu 502-0071, Japan

## Abstract

**Background:**

Agarwood (*Aquilaria sinensis*), well known as incense in Southeast Asia, has been used as a digestive in traditional medicine. We investigated the laxative effects of an ethanol extract of agarwood leaves (EEA) in a rat model of low-fiber diet-induced constipation.

**Methods:**

A set of rats was bred on a normal diet while another set was placed on a low-fiber diet to induce constipation. The laxative effect of agarwood was then investigated on both sets of rats.

**Results:**

Pretreatment of normal rats with single dose of EEA (600 mg/kg, p.o.) significantly increased frequency and weight of stools. Also, treatments with EEA (300 and 600 mg/kg, p.o.) for 14 days caused a significant increase in stool frequency and weight. Feeding of the animals with a low-fiber diet resulted in a decrease in stool weight, frequency, and water content and also delayed carmine egestion. A single treatment with EEA (600 mg/kg) or senna (150 and 300 mg/kg) significantly increased stool frequency, weight, and water content and also accelerated carmine egestion in the model rats. Once daily administrations of EEA (150 mg/kg), for 14 days, caused a significant increase in water content of stools. The higher doses of EEA (300 and 600 mg/kg) significantly increased frequency, weight, and water content of the stools while accelerating carmine egestion in the constipated rats. Senna (150 and 300 mg/kg) produced similar effect as the higher doses of EEA but, in addition, induced severe diarrhea.

**Conclusion:**

These findings indicate that EEA has a laxative effect, without causing diarrhea, in a rat model of low-fiber diet-induced constipation. These findings suggest that EEA may be highly effective on constipation as a complementary medicine in humans suffering from life style-induced constipation.

## Background

Constipation is a common public health problem with a well-recognized propensity to cause discomfort and to affect quality of life. Constipation increases during aging and can be a chronic condition requiring the use of laxatives over the long term. Constipation is not only discomforting but can also cause abdominal distension, vomiting, restlessness, gut obstruction, and perforation, and may be associated with aspiration or fatal pulmonary embolism [1]. At present, 20 to 30% of people over the age of 60 use a laxative more than once a week [2]. Drugs containing magnesium oxide or sennoside, the main constituent of senna, are typically administered for treatment of constipation due to their powerful purgative/laxative activities, but these drugs also induce severe diarrhea as a side effect. In addition, repeated use of senna or other anthraquinoide-containing drugs can induce melanosis coli (colic melanosis), a risk factor for colorectal neoplasm [3]. Agarwood, also well known as incense in Southeast Asia, has been used as a sedative, analgesic, and digestive in traditional medicine. Agarwood leaves are consumed as a healthy tea in Thailand and Taiwan. However, the botanical name "Agarwood" in English applies to more than fifteen different species (*Aquilaria apiculina*, found in the Philippines; *Aquilaria baillonil*, found in Cambodia; *Aquilaria baccarain*, found in Indonesia; *Aquilaria brachyantha*, found in Malaysia; *Aquilaria crasna*, found in Thailand, Malaysia and Cambodia; *Aquilaria sinensis*, found in Taiwan, etc.). In our previous study, we demonstrated that *Aquilaria sinensis *and *Aquilaria crasna *have laxative effects on a mouse model of loperamide-induced constipation, without inducing diarrhea. Agarwood (*Aquilaria sinensis*) also accelerates gastrointestinal transit in loperamide-induced constipation mouse model and increases the spontaneous contractions of isolated guinea pig jejunum and ileum. The increased contractions and laxative effect of agarwood can be blocked by atropine, a muscarinic receptor antagonist [4].

Constipation arises from a variety of causes (e.g., chemical compounds such as morphine, dietary habits, and psychological stress, etc.). The mouse model of loperamide-induced constipation corresponds to morphine-induced constipation in human patients since both loperamide and morphine are opioid-receptor agonists. In the present study, we used a rat model of low-fiber diet-induced constipation, which has the same similarities as human patients suffering from constipation as a result of poor dietary habits. The purpose of the present study was to investigate the laxative effects of EEA using a rat model of low-fiber diet-induced constipation.

## Methods

### Materials

Agarwood (*Aquilaria sinensis*, gathered in Daisoukou, Taipei, Taiwan) and senna (*Senna alexandrina*) leaves were supplied by API Co., Ltd. (Gifu, Japan). The species of these botanical materials were authenticated by Professor Munekazu Iinuma Ph.D. (Pharmacognosy, Department of Bioactive Molecules, Gifu Pharmaceutical University). The voucher specimen of EEA (JLE-50P-080924) was deposited at Nagara Research Center, API Co., Ltd. (692-3 Nagarayamasaki, Gifu 502-0071, Japan). Carmine was purchased from Wako Pure Chemical Co., Ltd. (Osaka, Japan).

### Extraction and isolation procedures

Agarwood and senna leaves (each 50 g) were chopped into smll pieces and extracted with 60% ethanol (1000 ml) at room temperature (25.0°C) for 24 h. We got 8.0-9.0 g of solid powder from each leaves every time.

### Animals

Male SD rats (6 weeks old) were purchased from Japan SLC (Hamamatsu, Japan). The animals were housed at a controlled room temperature (24.5-25.0°C) with a 12/12 h light/dark cycle. Food pellets (CE-2, CREA Japan, Inc., Tokyo, Japan) and tap water were provided *ad libitum*. All animal experiments were carried out according to the "*Principles of Laboratory Animal Care*" (NIH publication number 85-23, revised 1985) and "*Guidelines of the Animal Investigation Committee of Gifu Pharmaceutical University*".

### Ethical approval

All experiments were approved by the Animal investigation Committee of Gifu Pharmacological University.

### Stool parameters

**Figure 1:**
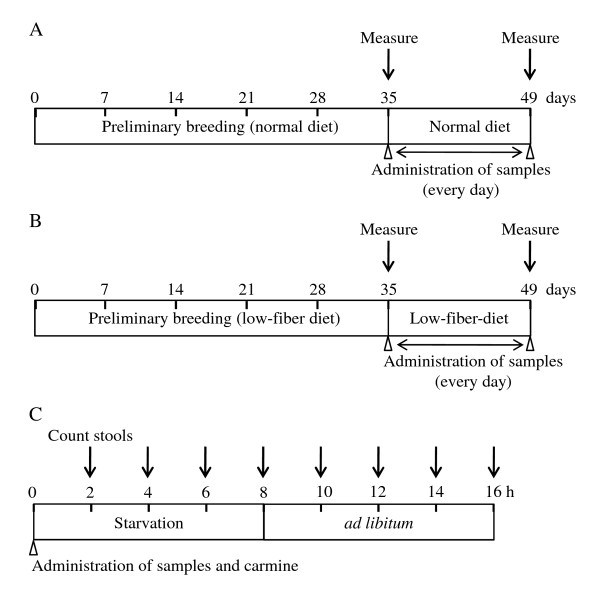
**The schedule of examination performed in this study**.

The frequency and weight of stools were measured as the frequency and total wet weight per rat over 16 h (Figure 1C). EEA (150, 300, and 600 mg/kg) and senna (150 and 300 mg/kg) were orally administered once daily for 14 days (Figure 1A). Gum arabic (5% w/w) was orally administrated as a vehicle. Carmine (10 mg/body) was administered *via *the same route immediately after sample administration. The frequency, weight, and water content of stools from each rat were measured at 2 h intervals for 16 hours (e.g. 0-2 h, 2-4 h, 4-6 h, etc.). Carmine egestion by each rat was measured at 2 h intervals for 24 h. Rats were then placed individually in stainless steel cage (24 × 38 × 20 cm) and fasted for 8 h but provided with water *ad libitum*.

### Induction and evaluation of constipation

Rats were maintained on a low-fiber diet for 5 weeks to induce constipation prior to the experiments (Figure 1B). The low-fiber diet (Crea Japan, Inc.) contained 41.5% cornstarch, 24.5% milk casein, 10.0% sucrose, 10.0% dextrin, 7.0% mineral mixture, 6.0% corn oil, and 1.0% vitamin mixture (Table 1). Induction of constipation were evaluated by stool parameters (frequency, weight, water contents, and carmine egestion).

**Table 1:** Composition of the normal diet and low-fiber diet.

	contents (%)	
	
Ingredients	normal diet	low-fiber diet
Moisture	9.3	9.0
Crude protein	25.1	21.9
Crude fat	4.8	6.1
Crude fiber	4.2	0.1
Crude ash	6.7	5.9
NFE	50.0	57.0

NFE: Nitrogen Free Extract

### Statistical analysis

Data are presented as mean ± S.E.M. Statistical comparisons were made with the Student's *t*-test, Tukey's multiple comparison test, or x^2 ^test (JSTAT for Windows; Vector, Tokyo, Japan).

## Results

### Laxative effects of EEA and senna in normal rats

**Figure 2:**
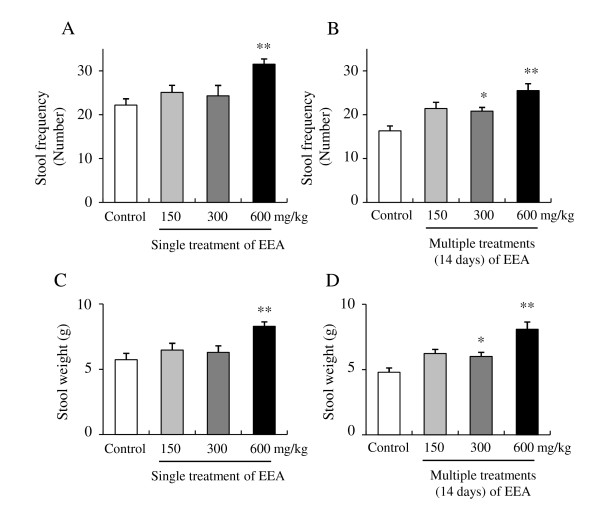
**Laxative effects of single (A and C) and 14-day (B and D) administration of EEA on normal rats**. Data are shown as the mean ± S.E.M., n = 12, *p < 0.05, **p < 0.01 vs. control (one-way ANOVA and Tukey's multiple comparison test).

To examine the laxative effects of EEA on frequency and wet weight of stools in normal male rats, EEA (150, 300, and 600 mg/kg) and gum arabic (as a control) were orally administered once daily for 14 days (Figure 1A). The frequency and the wet weight of stools were measured on the first day and again on the 14^th ^day (Figure 1C). The frequency and the wet weight of stools following a single administration of gum arabic were 22.2 ± 4.97 and 5.74 ± 1.70 g, respectively (Figure 2A and 2C). A single administration of EEA at 600 mg/kg significantly increased the frequency and wet weight of stools to 31.5 ± 4.25 and 8.29 ± 1.21 g, respectively (Figure 2A and 2C). A single administration of EEA at 150 and 300 mg/kg showed tendency to increase the frequency and wet weight of stools, but the increment was not statistically significant (Figure 2A and 2C).

The frequency and the wet weight of stools following multiple oral administrations of gum arabic for 14 days were 16.3 ± 3.98 and 4.79 ± 1.14 g, respectively (Figure 2B and 2D). Multiple oral administrations of EEA for 14 days significantly increased the frequency and wet weight of stools to 20.8 ± 2.98 and 6.00 ± 1.10 g, respectively, at 300 mg/kg or to 25.5 ± 5.42 and 8.09 ± 1.91 g, respectively, at 600 mg/kg (Figure 2B and 2D). A single administration of EEA at 150 mg/kg showed a tendency to increase the frequency and wet weight of stools, but the increment was not statistically significant (Figure 2B and 2D).

### The effect of low-fiber diet on stool parameters

**Figure 3:**
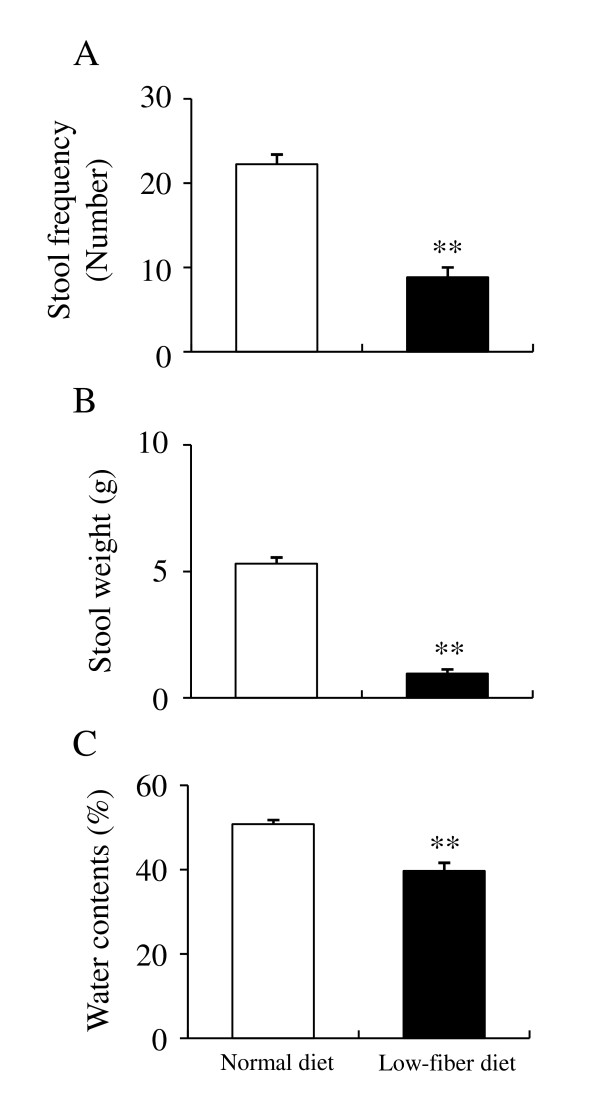
**Effects of normal diet (white bars) and low-fiber diet (black bars) on frequency (A) weight (B) and water content (C) of stools over a 16 h period in rats**. Data are shown as the mean ± S.E.M., n = 12, *p < 0.05, **p < 0.01 vs. vehicle (one-way ANOVA and Tukey's multiple comparison test).

Before assessing the laxative effects of EEA and senna on low-fiber diet-induced constipation in rats, the effects of a 5 week treatment with a low-fiber diet were determined on the frequency, weight, and water content of stools. Rats were purchased at 6 weeks old and maintained on a normal diet (as a control) or on a low-fiber diet for 5 weeks. After 5 weeks of feeding, carmine (10 mg/body) was orally administered, then stool frequency, weight, and water content were measured (Figure 3A, B, and 3C) and carmine egestion was investigated in each rat during consecutive 2-h periods for 16 h (Table 2). Stool frequency, weight, and water content of rats maintained on the normal diet were 22.3 ± 1.15, 5.31 ± 0.25 g, and 50.8 ± 1.00%, respectively (Figure 3A, B, and 3C). Treatment for 5 weeks with a low-fiber diet significantly reduced the stool frequency, weight, and water content to 8.83 ± 1.19, 0.97 ± 0.16 g, and 39.7 ± 1.92%, respectively, (Figure 3A, B, and 3C). The 5-week low-fiber diet also significantly delayed carmine egestion (Table 2).

**Table 2:** Effects of low-fiber diet on the rate of carmine egestion in rats.

		Number of rats egesting carmine-containing stools								
		
Treatments		0-2	2-4	4-6	6-8	8-10	10-12	12-14	14-16	over 16 h
Normal diet		-	-	-	-	-	5	4	3	-
Low-fiber diet	**	-	-	-	-	-	-	-	-	12

Rats were maintained on a normal-diet or a low-fiber diet for 5 weeks. Carmine (10 mg/body) was orally administered to the rats, then the weights of carmine-containing-stools were measured during consecutive 2-h periods over 16 h. n = 12, **p < 0.01 vs. normal-diet (Chi-square test).

### Effects of EEA and senna on stool parameters in the rat model of low-fiber diet-induced constipation

**Figure 4:**
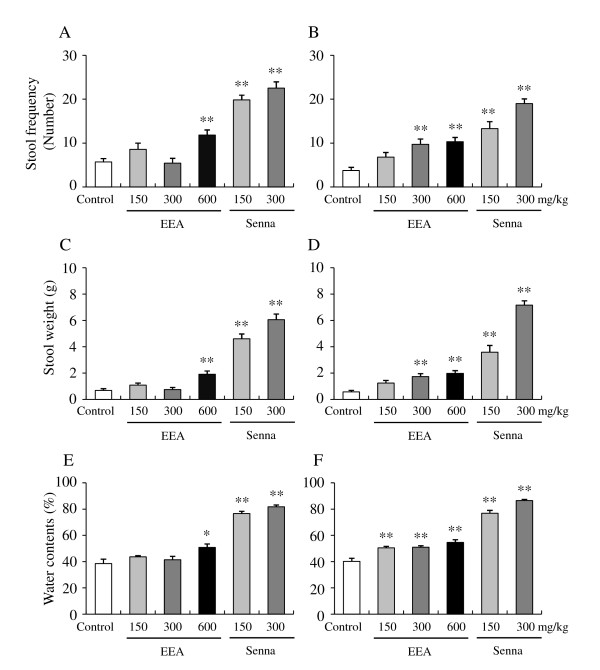
**Effect of single (A, C and E) and 14-day (B, D and F) administrations of EEA and senna on stool frequency, weight and watercontent on rats with low fiber diet-induced constipation**. Data are shown as the mean ± S.E.M., n = 12, **p < 0.01, *p < 0.05 vs. vehicle (paired Tukey's multiple comparison test).

To examine the effects of EEA and senna on stool frequency, weight, and water content and on carmine egestion in the low-fiber fed rats, constipation was induced in the rats by maintaining them on the low-fiber diet for 5 weeks. Then, EEA (150, 300, and 600 mg/kg), senna (150 and 300 mg/kg), and gum arabic (as a control) were orally administered once daily for 14 days according to the schedule shown (Figure 1B). Stool frequency, wet weight, and water content following a single administration of gum arabic were 5.70 ± 2.74, 0.70 ± 0.40 g, and 38.6 ± 11.7%, respectively (Figure 4A, C, and 4E). A single administration of EEA at 600 mg/kg significantly increased stool frequency, wet weight, and water content to 11.8 ± 4.04, 1.90 ± 0.86 g, and 50.9 ± 8.9%, respectively (Figure 4A, C, and 4E). A single administration of EEA at either 150 or 300 mg/kg did not affect stool frequency, wet weight, or water content (Figure 4A, C, and 4E).

A single administration of senna significantly increased stool frequency, wet weight, and water content to 19.8 ± 3.76, 4.6 ± 1.28 g, and 76.6 ± 6.1% respectively, at 150 mg/kg and to 22.5 ± 5.05, 6.10 ± 1.47 g, and 81.8 ± 4.70%, respectively, at 300 mg/kg (Figure 4A, C, and 4E). A single administration of EEA at 600 mg/kg and of senna at 150 and 300 mg/kg significantly accelerated carmine egestion (Table 3A). A single administration of senna at 150 or 300 mg/kg induced diarrhea in all the animals used (Table 4).

**Table 3:** Effects of EEA and senna on carmine egestion in a rat model of low-fiber diet-induced constipation.

A) Single administration													
		**Number of rats egesting carmine-containing stools over 1g**											
		
**Treatments**	**mg/kg**	**0-2**	**2-4**	**4-6**	**6-8**	**8-10**	**10-12**	**12-14**	**14-16**	**16-18**	**18-20**	**20-22**	**22-24 h**

Control		-	-	-	-	-	-	-	1	-	2	2	7
EEA	150	-	-	-	-	-	-	2	-	-	1	5	4
EEA	300	-	-	-	-	-	-	-	1	1	1	7	2
EEA	600*	-	-	-	-	-	4	2	2	2	-	1	1
Senna	150**	-	-	5	3	3	-	1	-	-	-	-	-
Senna	300**	-	1	6	4	1	-	-	-	-	-	-	-

**B) Multiple administrations for 14 days**													

		**Number of rats egesting carmine-containing stools over 1g**											
		
**Treatments**	**mg/kg**	**0-2**	**2-4**	**4-6**	**6-8**	**8-10**	**10-12**	**12-14**	**14-16**	**16-18**	**18-20**	**20-22**	**22-24 h**
Control		-	-	-	-	-	-	-	-	1	-	1	10
EEA	150	-	-	-	-	-	-	1	-	-	3	1	7
EEA	300*	-	-	-	-	-	-	-	3	1	4	-	4
EEA	600**	-	-	-	-	-	1	2	2	3	3	1	-
Senna	150**	-	-	1	3	3	2	1	-	-	-	1	1
Senna	300**	-	5	5	2	-	-	-	-	-	-	-	-

Low-fiber diet-induced constipation was induced in rats by feeding a low fiber diet for 5 weeks. EEA (150, 300, and 600 mg/kg) and senna (150 and 300 mg/kg) were orally administered daily for 14 days, and carmine (10 mg/body) was orally administered to rats just after the administration of the EEA or senna. Effects of single administration of EEA on carmine egestion (A). Effects of multiple oral administrations of EEA on carmine egestion (B). n = 12, **p < 0.01, *p < 0.05 vs. control (Chi-square test).

**Table 4:** Effects of EEA and senna on diarrhea frequency.

		Frequency of diarrhea (%)		
		
Treatments	Dose (mg/kg)	Day 1	Day 7	Day 14
Control		0	0	0
EEA	150	0	0	0
EEA	300	0	0	0
EEA	600	0	8.3	0
Senna	150	100	100	91.7
Senna	300	100	100	100

EEA (150, 300, and 600 mg/kg) and senna (150 and 300 mg/kg) were orally administered daily to constipated rats that had been maintained on a low-fiber diet for 5 weeks. EAA or senna were orally administered to the rats, then the frequency of diarrhea was measured over the next 16 h. n = 12.

Stool frequency, wet weight, and water content following multiple oral administrations of gum arabic for 14 days were 3.75 ± 2.53, 0.57 ± 0.42 g, and 40.2 ± 7.7%, respectively (Figure 4B, D, and 4F). Multiple oral administrations of EEA for 14 days significantly increased stool frequency, wet weight, and water content to 9.7 ± 4.3, 1.73 ± 0.79 g, and 51.0 ± 4.0%, respectively, at 300 mg/kg and to 10.3 ± 3.4, 1.98 ± 0.73 g, and 54.7 ± 7.0%, respectively, at 600 mg/kg (Figure 4B, D, and 4F). Multiple oral administrations of EEA at 150 mg/kg significantly increased stool water content to 50.5 ± 4.2 (Figure 4F), but did not affect either the stool frequency or stool weight (Figure 4B and 4D).

Multiple oral administrations of senna significantly increased the frequency, wet weight, and water content of stools to 13.3 ± 5.5, 3.59 ± 1.77 g, and 76.9 ± 7.6%, respectively, at 150 mg/kg and to 19.0 ± 3.7, 7.16 ± 1.14 g, and 86.5 ± 3.2%, respectively, at 300 mg/kg (Figure 4B, D, and 4F). Multiple oral administrations of EEA at 300 and 600 mg/kg and of senna at 150 and 300 mg/kg also significantly accelerated carmine egestion (Table 3B). Multiple oral administrations of senna at 150 and 300 mg/kg also induced diarrhea at the rates of 91.7 and 100%, respectively (Table 4).

## Discussion

In the rats fed normal diets, a single administration of EEA at 600 mg/kg significantly increased stool frequency and weight. EEA at 150 and 300 mg/kg showed a tendency to increase the frequency and weight of stools, but this difference was not statistically significant. Multiple oral administrations of EEA at 300 and 600 mg/kg once daily for 14 days significantly increased stool frequency and weight. EEA at 300 mg/kg showed significant efficacy in normal mice at multiple administrations for 14 days, but was not effective as a single administration. These findings indicate that laxative effect of EEA on constipation is increased by dosage when supplied as a single administration and that multiple administrations of EEA do not induce tolerance.

Herbal medicines are now used by up to 50% of the Western population and they are used at least in part for the treatment or prevention of digestive disorders. Although most indications for the use of herbal medicines are anecdotally or traditionally derived, some of their efficacies have been scientifically demonstrated. Any herbal medicine may contain many bioactive compounds with potentially deleterious as well as beneficial effects [5]. Senna, a common herbal medicine, is frequently used as both an ethical pharmaceutical and as a proprietary drug. The deleterious effects of senna are severe diarrhea and abdominal cramping [5]. Therefore, senna is unsuitable for daily use.

Agarwood (*Aquilaria sinensis *etc.) is consumed as a traditional medicine (in the form of a tea) in regions of Southeast Asia, including Thailand, Taiwan, and Cambodia. Our previous report showed that an acetone extract of agarwood had a laxative effect on normal mice and that the main pharmacologically active constituents of this extract is genkwanin-5-*O*-beta-primeveroside. When purified, genkwanin-5-*O*-beta-primeveroside increased the intestinal contractions of the ileum isolated from guinea pigs *via *action on acetylcholine receptors [6].

The actual botanical species called "agarwood" varies depending on region and is represented by more than fifteen different species [7]. In the previous study, we focused on two species (*Aquilaria sinensis*; from Taiwan, and *Aquilaria crasna*; from Thailand) and demonstrated that ethanol extracts of from either "agarwood" species had a laxative effect equal to that of the acetone extract of *Aquilaria sinensis *in a mouse model of loperamide-induced constipation [4]. We also demonstrated that an ethanol extract of *Aquilaria sinensis *increases both the intestinal tension of the small intestine and the rate of gastrointestinal transit.

In the present study, we demonstrated the laxative effects of EEA (*Aquilaria sinensis*) in a rat model of low-fiber diet-induced constipation. Maintaining a low-fiber diet for 5 weeks significantly decreased stool frequency, weight, and water content and significantly delayed carmine egestion. A single administration of EEA at 600 mg/kg significantly increased stool frequency, weight, and water content and significantly accelerated carmine egestion. Multiple administrations of EEA at 300 and 600 mg/kg also significantly increased the frequency and weight of stools, and at 150-600 mg/kg, multiple administrations significantly increased stool water content and the rate of carmine egestion. A single treatment of EEA at 150 and 300 mg/kg did not show efficacy, but repeated treatment of EEA at 300 mg/kg showed a significant increase in stool weight and stool frequency. These results indicate that EEA ameliorated low-fiber diet-induced constipation in rats; therefore, EEA may be suitable for human patients suffering from constipation due to their diet style.

Low-fiber diet-induced constipation relates to fecal consistency, because fecal consistency depends on the ratio of the water-holding capacity of the insoluble solids, such as dietary fibers. When there are sufficient water-holding solids and/or little non-bound (free) water, stools remain thick or formed. On the other hand, if there are too few of these water-holding solids to bind all of the water present, stool consistency becomes loose, eventually to the point of being like water [8]. Repeated treatment of EEA at 150 mg/kg significantly improved the water contents, though failed to improve stool weight and stool frequency. This result indicates that compensation of water contents is not sufficient to show laxative activity in low-fiber diet-induced constipation.

Intestines of low-fiber diet-induced constipation animal are not inhibited by chemical constituents such as loperamide or clonidine. As our previous report showed, genkwanin-5-*O*-beta-primeveroside could play a key role in laxative activity *via *acetylcholine receptors in the presented low-fiber diet-induced model.

Laxative effect can also be exhibited by an increase in secretions in the GI tract, for e.g., like castor oil. This type of laxatives induce diarrhea, because secreted material liquefy stool consistency. EEA may have such effect, but its effect is not main mechanism of the laxative effect, because EEA does not induce the diarrhea.

EEA and senna both accelerated carmine egestion and these results indicate that EEA and senna shortened the preservation time of food in the digestive tract. On the other hand, in contrast to senna, orally administered EEA did not induce diarrhea, nor did it affect body weight (data not shown). Our previous report demonstrated that EEA increases contractions of the jejunum and ileum isolated from guinea pigs [7]. Collectively, these findings indicate that EEA may increase contractions of the intestine to accelerate food in the digestive tract without inhibiting nutrient absorption.

## Conclusions

In conclusion, EEA, administered either in a single dose or multiple doses, showed a laxative effect on rats with low-fiber diet-induced constipation without causing the diarrhea typical of senna administration. These findings suggest that EEA may be useful as a therapeutic laxative agent in humans.

## List of abbreviation used

EEA: Ethanol extract of agarwood leaves

## Competing interests

The agarwood leaves were supplied by API Co., Ltd. (Gifu, Japan). There is no other competing interest.

## Authors' contributions

MK mainly performed the study, analyzed the data, and wrote the paper; ST supported the study, prepared the samples, and participated in the design of the study; HM, KT, YA, and MS participated in the design of the study and acquisition of the samples; HH conceived and organized the study, participated in the design of the study, and acquisition of the samples. All authors read and approved the final manuscript.

## Pre-publication history

The pre-publication history for this paper can be accessed here:

http://www.biomedcentral.com/1472-6882/10/68/prepub
